# Palliative radiotherapy with or without additional care by a multidisciplinary palliative care team in patients with newly diagnosed cancer: a retrospective matched pairs comparison

**DOI:** 10.1186/s13014-015-0365-0

**Published:** 2015-03-07

**Authors:** Carsten Nieder, Astrid Dalhaug, Adam Pawinski, Ellinor Haukland, Bård Mannsåker, Kirsten Engljähringer

**Affiliations:** Department of Oncology and Palliative Medicine, Nordland Hospital, Bodø, 8092 Norway; Institute of Clinical Medicine, Faculty of Health Sciences, University of Tromsø, Tromsø, 9037 Norway

**Keywords:** Palliative care, Palliative radiotherapy, Palliative team, Supportive care

## Abstract

**Purpose:**

To analyze survival after early palliative radiotherapy (RT) in patients managed exclusively by regular oncology staff or a multidisciplinary palliative care team (MPCT) in addition.

**Methods:**

Retrospective matched pairs analysis. Comparison of two groups of 29 patients each: MPCT versus none. Early RT started within three months after cancer diagnosis.

**Results:**

Bone and brain metastases were common RT targets. No significant differences in baseline characteristics were observed between both groups. Twelve patients in each group had non-small cell lung cancer. Median performance status was 2 in each group. Twenty-seven patients in each group had distant metastases. Median survival was not significantly different. In multivariate analysis, MPCT care was not associated with survival, while performance status and liver metastases were. Rate of radiotherapy during the last month of life was comparable. Only one patient in each group failed to complete radiotherapy.

**Conclusions:**

MPCT care was not associated with survival in these two matched groups of patients. The impact of MPCT care on other relevant endpoints such as symptom control, side effects and quality of life should be investigated prospectively.

## Background

Patients with advanced cancer often experience considerable burden from symptoms such as pain, reduced mobility, dyspnea, cough, bleeding or neurological deficits, which may improve after palliative radiotherapy [1,2]. Given the complexity of symptoms and possibilities for pharmacological and other interventions, additional expertise from different health care professions might improve the overall benefit in terms of symptom control, management of side effects and quality of life (QoL). A recent retrospective analysis suggested that involving a multidisciplinary palliative care team (MPCT) in addition to regular oncology staff was associated with reduced likelihood of incomplete palliative radiotherapy, although the observed difference was not statistically significant [3]. In multivariate analysis, MPCT care was not associated with survival. Most patients were treated late during the disease trajectory.

In contrast, a randomized trial of early palliative care, limited to patients with newly diagnosed metastatic non-small cell lung cancer (NSCLC) and not tied to radiotherapy utilization, showed marked differences [4,5]. Participants (n = 151) were recruited at a single institution during the time period between 2006 and 2009. Early palliative care integrated with standard oncology care was compared to standard oncology care alone. Patients assigned to the experimental arm consulted with a member of the MPCT within 3 weeks of enrollment and at least monthly thereafter. Those assigned to the standard care arm only met with the MPCT on request from the patient, family, or oncologist. Early palliative care integrated with standard oncologic care led to significant improvements in QoL and mood from baseline to 12 weeks. Fewer patients received aggressive end-of-life care, yet median survival was longer among patients receiving early palliative care (11.6 versus 8.9 months).

Due to these observations and the heterogeneity of the patient group included in our previous study, we were interested in analyzing the impact of a MPCT on survival after palliative radiotherapy, use of radiotherapy near the end of life, and successful completion of fractionated treatment in patients treated early after diagnosis, arbitrarily defined as the first three months from histological confirmation of the malignant disease. In order to further reduce bias, MPCT patients were matched to standard care patients based on established prognostic factors for survival, such as cancer type and performance status (PS). Compared to our initial study, we extended the inclusion period from 2009 to 2012.

## Methods

We performed a retrospective analysis, using a database in which all patients treated with palliative radiotherapy at our hospital are registered. The patients started their treatment in the time period from June 20, 2007 (date of opening of the hospital’s radiotherapy facility) to December 31, 2012. From the database we identified all patients who received early palliative radiotherapy, i.e. within three months from cancer diagnosis (n = 148). The target volumes included distant metastases, lymph node metastases or primary tumors. Due to their different biological behavior, hematological malignancies and primary central nervous system tumors were not included. Stereotactic radiotherapy was not available. Typical fractionation regimens were 3 Gy x10, 4 Gy x5 or 8 Gy x1. Then we used the hospital’s electronic patient record (EPR) system to determine whether standard oncology care or additional care by our MPCT was provided. In many cases, the MPCT was already involved before referral to radiotherapy, but we also included patients with simultaneous start of radiotherapy and MPCT care.

Referral to the MPCT was not standardized. Rather, individual decisions were made by the treating clinical oncologists responsible for chemo- and radiotherapy delivery, based on symptom severity, pain control or need for initiation of home care services, taking into account patient preferences. Our MPCT staff, which collaborates closely with primary health care facilities, family doctors and home care providers in the region, includes several professions: physician, nurse, psychologist, physiotherapist, nutritionist, and priest. Regular weekly meetings between clinical oncologists and MPCT took place. Both MPCT and radiotherapy facility operated every workday. Time from referral to MPCT to first appointment was 1-2 days. All patients were covered by the national public insurance system. Therefore, no out-of-pocket costs were incured for any patient, regardless of management approach/treatment intensity. In other words, no particular barriers prevented patients from access to the MPCT.

Overall, 29 patients (20%) had received MPCT care in addition to radiotherapy. Each patient was matched to one patient managed with standard care not involving the MPCT. The investigator that performed the matching had access to baseline data, but was blinded to outcomes. Identical Eastern Cooperative Oncology Group (ECOG) PS and stage of disease (distant metastases or no distant metastases) was required. Furthermore, age (<10 years difference) and natural course of disease had to be comparable (favorable biology vs. unfavorable biology). The latter was based on a diagnosis of breast/prostate cancer vs. lung cancer vs. other primary tumors. If several matches were possible, systemic therapy was added as selection parameter (ongoing systemic therapy vs. none). If still more than one patient fulfilled these criteria, the one treated in the closest temporal relationship to the MPCT patient was chosen.

Radiation treatment details and date of death were available from the EPR system. All patients had died by the time of this analysis. Survival time was measured from day 1 of radiotherapy. Actuarial survival curves were generated by Kaplan-Meier method and compared by log-rank test (IBM SPSS Statistics 21 (IBM Corporation, Armonk, NY, USA)). Prognostic factors achieving statistical significance (defined as p < 0.05 throughout this study in two-sided tests) were entered into multivariate survival analysis (Cox regression). Univariate analyses of baseline parameters consisted of Pearson chi-square and Fisher’s exact test.

We assessed the statistical fundament of this study, assuming a mean survival time of 6 months (standard deviation 4 months) in the non-MPCT group, as reported previously [3]. With 29 patients per group, the potential survival improvement in the MPCT group would have to be 2.6 months (mean 8.6 ± 4 months) if one requires 80% statistical power and 5% alpha error level.

The study was performed as a retrospective analysis of early palliative radiotherapy. As a quality of care analysis, no approval from the Regional Committee for Medical and Health Research Ethics (REK) was necessary.

## Results

The characteristics of the two groups are shown in Table 1. Bone metastases (n = 11 in each group) and brain metastases (n = 9 in each group) were common radiotherapy targets. Regarding primary cancer type, the largest subgroup was the one with NSCLC (n = 12 in each group). Virtually all patients had distant metastases (n = 27 in each group). Median ECOG PS was 2 in both groups. Median age was 65 and 67 years, respectively. No statistically significant differences in baseline characteristics were found.

**Table 1:** **Patient characteristics**

**Univariate analysis of baseline parameters for patients treated with early palliative RT with or without care by multidisciplinary palliative care team (MPCT)**			
**Parameter**	**Without MPCT**	**With MPCT**	**p** ***-*** **Value**
	**Number of patients**	**Number of patients**	
Patient number	29	29	
ECOG performance status			
0/1	8	8	
2	11	11	
3/4	10	10	1.0
Median age at RT (years, range)	65 (51-82)	67 (42-81)	0.83
Gender			
Male	14	15	
Female	15	14	1.0
Primary tumor site			
Prostate/Breast	1	1	
Lung (small cell)	3	2	
Lung (non-small cell)	12	12	
Kidney	7	7	
Others	6	7	0.83
Total no. of TV in RT course			
1	19	19	
2	7	8	
3	3	2	0.88
Disease extent			
Distant metastases	27	27	
No distant metastases	2	2	1.0
Liver metastases			
No	24	21	
Yes	5	8	0.53
Lung metastases			
No	17	21	
Yes	12	8	0.41
Adrenal gland metastases			
No	26	22	
Yes	3	7	0.30
Brain metastases			
No	20	20	
Yes	9	9	1.0
Bone metastases			
No	11	12	
Yes	18	17	1.0
Metastatic spinal cord compression			
No	26	26	
Yes	3	3	1.0
Steroids during RT^a^			
No	10	7	
Yes	19	22	0.36
Analgetics^a^			
No opioids	12	10	
Opioids	17	19	0.79
Systemic cancer treatment			
No	23	22	
Started before RT	6	7	1.0
Charlson comorbidity index			
0	8	4	
1-2	10	16	
>2	11	9	0.23
Incomplete RT^b^			
No	25	26	
Yes	1	1	1.0

*RT* Radiotherapy, *TV* Target volume.

^a^information collected from patient charts rather than pharmacy databases.

^b^excluding single fraction RT, which always was completed.

**Figure 1:**
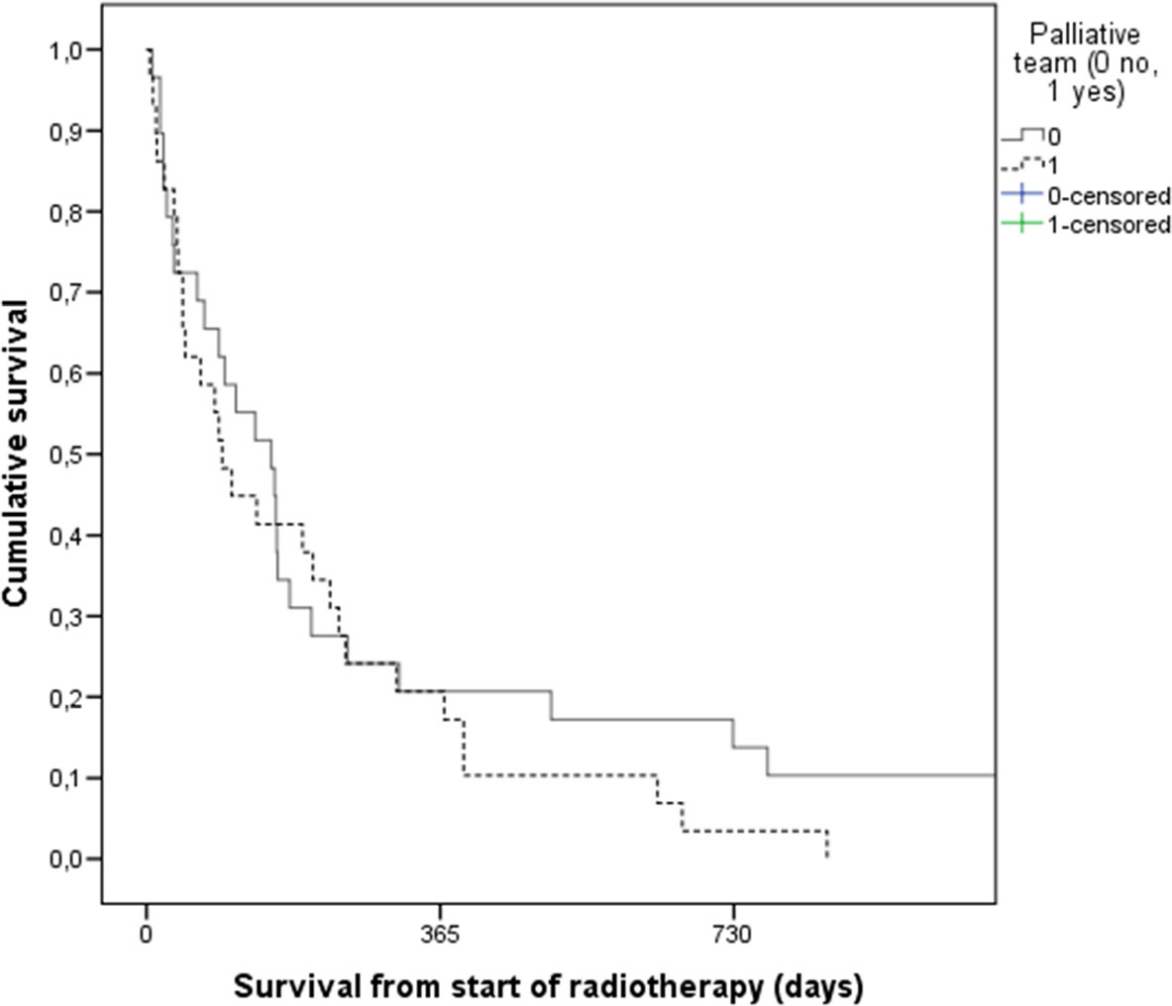
**Actuarial overall survival after early palliative radiotherapy (Kaplan-Meier estimate).** Median 94 days in the group managed by MPCT and 155 days in the group managed with standard care, p = 0.35.

Failure to complete radiotherapy was similarly uncommon in both groups (1 patient each). Median survival was not significantly different, 94 days with MPCT and 155 days without MPCT, p = 0.35 (Figure 1). Two prognostic factors were significantly associated with survival in multivariate analysis: better ECOG PS (p = 0.001) and absence of liver metastases (p = 0.04). Neither other baseline characteristics nor MPCT care predicted survival in the Cox model. Rate of radiotherapy during the last month of life was 28% in patients without MPCT and 21% in patients with MPCT, p = 0.76).

## Discussion

This study was designed as an expansion of our previous work, which had demonstrated a numerically lower rate of incomplete radiotherapy in patients managed by MPCT in addition to regular staff [3]. Survival was not significantly different. That study also revealed major imbalances between the two groups, suggesting that MPCT patients had more advanced disease, poorer PS and larger symptom burden. In general, MPCT support started quite late during the disease trajectory. Survival of patients with metastatic breast, prostate or kidney cancer is currently measured in years rather than months [6-8]. A thought-provoking randomized study suggested that early palliative care might be preferable over standard oncology care [4,5]. The authors even reported improved survival in their setting of NSCLC therapy and consultation with the MPCT within 3 weeks of enrollment. However, this was not the primary study endpoint and might or might not have resulted from imbalances in patient characteristics. All these findings, especially the difference in survival, inspired us to follow-up on our initial work and compare well-matched groups of patients treated earlier during the course of disease. Surprisingly, our choice of 3 months from cancer diagnosis resulted in identification of a rather small group of 29 patients. Narrowing this time interval further, e.g. to 3-4 weeks, would render this analysis meaningless, because group size and statistical power would become unacceptable. Actually, early treatment was not as early as anticipated, considering the fact that more than 20% of the patients were found to have received radiotherapy during the last month of life, i.e. during terminal illness. Much lower rates were found in previous analyses of patients who received radiotherapy at any point during the disease trajectory [9-12]. Our data suggest that patients referred to palliative radiotherapy soon after cancer diagnosis represent a prognostically unfavorable group.

It is noteworthy that few patients had PS 0-1 or biologically favorable cancer types, while most had metastatic disease. Use of steroids and opioid analgetics was common. These findings underline that early radiotherapy (most patients had not yet started systemic therapy when radiotherapy commenced) is not synonymous to limited disease extent or symptom burden. Obviously there was a reason for referral to the MDCT early after diagnosis in these 29 patients. As mentioned before, MDCT care was not standard at our institution. Rather, individual assessment was performed. This process is relatively subjective, as emphasized by the fact that we could match patients with comparable characteristics who were not managed by our MDCT. The matching aimed at elimination of important sources of bias. However, residual differences regarding prognostic factors such as weight loss/cachexia, anemia and others still might have influenced the results. Perfectly balanced groups cannot be created from small databases. Further drawbacks also exist: MPCT referral was not standardized, patients from the no-MPCT group might have had contact with the team at later time points after radiotherapy was completed, statistical power was limited, symptom severity and improvement were not assessed. Clearly, our study cannot replace a sufficiently large randomized prospective trial. Strengths of our study include the amount of baseline information and completeness of follow-up.

As shown in the results section, we were not able to demonstrate associations between MPCT care and overall survival or radiotherapy utilization near the end of life. The MPCT group had numerically shorter median survival, likely because only the sicker patients were referred. This might have caused imbalances in prognostic factors, e.g. weight loss or dyspnea. Few patients were unable to complete their prescribed course of radiotherapy, probably because hypofractionated regimens tailored to the expected prognosis were used. Other advantages of MPCT care still might exist. As demonstrated by Temel et al. these are measurable and clinically important [4]. It is also possible that survival improvement only can be observed in prognostically favorable patients, such as those included in the NSCLC trial [4], and that our patients who were in need of radiotherapy, represented a negative selection. MPCTs have been established in many hospitals with radiotherapy facilities, and such teams often provide additional supportive care interventions, physical exercise and therapy, and spiritual care, focusing on patients and caregivers [13-15]. Pituskin et al. reported on multidisciplinary assessment of patients with symptomatic bone metastases attending a dedicated outpatient palliative radiotherapy clinic [16]. Consecutive patients were screened for symptoms and needs relevant to their medications, nutritional intake, activities of daily living, and psychosocial and spiritual concerns from January 1 to December 31, 2007. Consultations by appropriate team members and resulting recommendations were collected prospectively. Patients who received radiotherapy were contacted by telephone four weeks later to assess symptom outcomes. A total of 106 clinic visits by 82 individual patients occurred. In addition to pain relief, significant improvements in tiredness, depression, anxiety, drowsiness and overall well-being were reported at four weeks.

## Conclusions

Increasing evidence suggests that MPCTs play an important role in the multidisciplinary management of patients with incurable cancer. Our data do not support the hypothesis that this type of care also improves survival, at least in the context of palliative radiotherapy.
